# Comprehensive Review of AL amyloidosis: some practical recommendations

**DOI:** 10.1038/s41408-021-00486-4

**Published:** 2021-05-18

**Authors:** Rama Al Hamed, Abdul Hamid Bazarbachi, Ali Bazarbachi, Florent Malard, Jean-Luc Harousseau, Mohamad Mohty

**Affiliations:** 1 Department of Internal Medicine, Jacobi Medical Center, Albert Einstein College of Medicine, New York, NY USA; 2 Department of Internal Medicine, American University of Beirut, Beirut, Lebanon; 3 Department of Clinical Hematology and Cellular Therapy, Saint-Antoine Hospital, AP-HP, Sorbonne University, and INSERM, Saint-Antoine Research Centre, Paris, France; 4 Institut de Cancerologie de l’Ouest, Centre René Gauducheau, Nantes-St Herblain, France

**Keywords:** Haematological diseases, Immunotherapy, Combination drug therapy

## Abstract

Amyloid light chain (AL) amyloidosis is among the more common and more severe of the amyloidoses usually involving the slow proliferation of a bone-marrow-residing plasma cell (PC) clone and the secretion of unstable immunoglobulin-free light chains (FLC) that infiltrate peripheral tissues and result in detrimental end-organ damage. Disease presentation is rather vague, and the hallmark of treatment is early diagnosis before irreversible end-organ damage. Once diagnosed, treatment decision is transplant-driven whereby ~20% of patients are eligible for autologous stem cell transplantation (ASCT) with or without bortezomib-based induction. In the setting of ASCT-ineligibility, bortezomib plays a central role in upfront treatment with the recent addition of daratumumab to the current emerging standard of care. In general, management of AL amyloidosis is aimed at achieving deep, durable responses with very close monitoring for early detection of relapse/refractory disease. This article provides a comprehensive review of the management of patients with AL amyloidosis including goals of therapy, current treatment guidelines in the setting of both ASCT-eligibility and ineligibility, treatment response monitoring recommendations, toxicity management, and treatment of relapse/refractory disease.

## Introduction

Systemic amyloidoses constitute disorders of diverse etiologies involving the synthesis and abnormal extracellular deposition of misfolded proteins in various organs with resultant damage. Among the different amyloidoses, amyloid light chain (AL), previously called “primary”, amyloidosis is among the more common and more severe, affecting ~10 per million per year^1^. Even though rarely due to a nonplasma cell B-cell close, AL amyloidosis is usually a plasma cell (PC) disorder whereby a small, slowly proliferating, bone-marrow-residing PC clone, secretes unstable immunoglobulin light chains. Those amyloidogenic free light chains (FLCs) can then infiltrate peripheral organs resulting in organ dysfunction and ultimately, failure. Organs involved usually include kidneys, heart, gastrointestinal (GI) tract, liver, and nervous system whereby undoubtedly cardiac involvement is the main driver of disease prognosis and mortality^2^. Given that more than 69% of patients already have more than one organ involved at the time of diagnosis^3^, it becomes vital to not only diagnose AL amyloidosis early, but to also effectively control plasma cell dyscrasias and thus halt escalating organ damage.

Owing to toxicity, autologous stem cell transplantation (ASCT) remains the standard of care and first-line treatment in a small proportion of patients. Importantly, the outcome of ASCT-ineligible patients has improved by the introduction of new first-line agents, specifically bortezomib. Currently, active research involving second-line novel treatments and anti-CD38 antibodies offers new hope. Nonetheless, the main hallmark of management remains early recognition and initiation of treatment before the occurrence of irreversible organ damage.

It becomes vital to differentiate AL amyloidosis from transthyretin (TTR) amyloidosis, another disease of increasing prevalence, due to the difference in management. TTR is a liver-synthesized thyroxine and vitamin A transporter^4,5^. Age (wild-type, previously called senile amyloidosis) or an autosomal dominant amino acid substitution (mutant) result in fibrillogenesis whereby TTR dissociates into intermediates that misassemble into amyloid fibrils and deposit in end-organs, most notably the heart^6^. While AL amyloidosis is treated with chemotherapy and transplant, TTR cardiac amyloidosis is treated with targeted therapy such as tafamidis^7^.

## Diagnostic approach

**Fig. 1:**
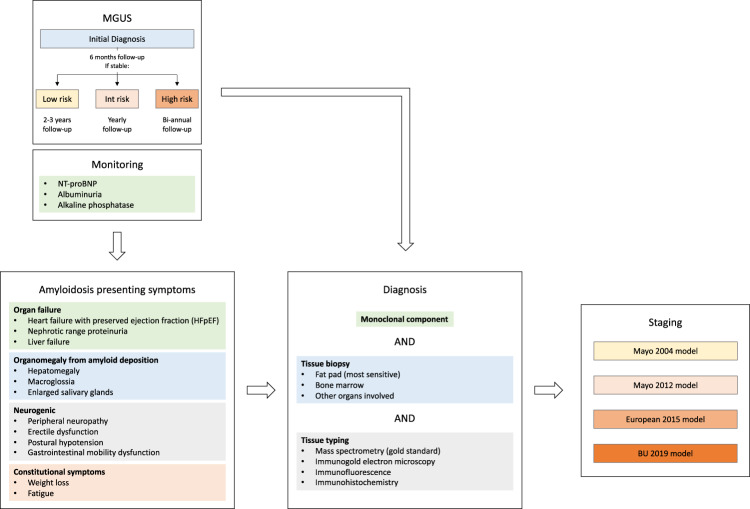
Monitoring, presenting symptoms, anddiagnosis of AL amyloidosis. MGUS monoclonal gammopathy of undetermined significance, NT-proBNP N-terminal pro b-type natriuretic peptide.

The symptoms and the presentation of amyloidosis depend on the organs involved in the disease and so, patients can present with a myriad of unspecific symptoms that could be easily misinterpreted, thus clouding the diagnosis of amyloidosis and delaying treatment initiation. Common symptom constellations include, but are not limited to, heart failure with preserved ejection fraction (HFpEF), nephrotic range proteinuria, organomegaly due to amyloid deposition (hepatomegaly, macroglossia, enlarged salivary glands etc.), peripheral neuropathy, and constitutional symptoms (weight loss, fatigue) (Fig. 1). By the time such symptoms surface, organ damage has already occurred. It thus becomes important to identify high-risk patient populations who would benefit from regular screening to establish a diagnosis before symptom onset. This includes monoclonal gammopathy of undetermined significance (MGUS) patients who would benefit from regular follow-up of markers such as N-terminal pro-brain natriuretic peptide (NT-proBNP) and albuminuria, which could begin to rise before overt HF and nephrotic syndrome develop^8,9^ (Fig. 1). Once suspected and a monoclonal component is confirmed, confirmation requires tissue biopsy (fat pad, bone marrow (BM), salivary gland, involved organ) and typing (mass spectrometry [current gold standard], immunogold electron microscopy, immunofluorescence, and immunohistochemistry) followed by risk stratification and disease staging^9^ (Fig. 1).

There are four staging models that utilize cardiac soluble biomarkers—Mayo 2004 model, European 2015 modification of Mayo 2004 model, Mayo 2012 model, and Boston University (BU) model^10–14^ (Table 1). The Mayo 2004 model, uses troponin T (TnT) and NT-proBNP and categorizes disease into one of three stages according to the specified thresholds of TnT <;0.035 microg/L and NT-proBNP < 332 ng/L^11^. Depending on whether TnT and NT-proBNP were both low, only one was high or both were elevated, disease was classified as stage I, II, or III, respectively^11^. In 2015, a European group proposed a modification of the Mayo 2004 model, in which patients classified in stage III of the Mayo model were further subclassified into low-risk and high-risk groups, stage IIIA and IIIB, using a new threshold for NT-proBNP of 8500 ng/L. Patients with an NT-proBNP > 8500 ng/L had higher risk disease and poorer prognosis^12^. The Mayo model was revised in 2012 incorporating different thresholds for TnT and NT-proBNP and introducing difference in FLC (dFLC) as an additional marker for disease burden^13^. One point was attributed to each of TnT ≥ 0.025 ng/mL, NT-proBNP ≥ 1800 pg/mL, and dFLC ≥ 18 mg/dL, thus classifying disease into four stages, I–IV according to a total score of 0–3, respectively^13^. Recently, the BU group derived a new staging system that correlates with the Mayo 2004 system, but utilizes BNP instead^14^. A BNP threshold >81 pg/mL best identified cardiac involvement and thus correlated with the Mayo 2004 staging system (κ = 0.854)^14^. Three stages were thus developed based on a BNP > 81 pg/mL and troponin I (TnI) > 0.1 ng/mL, whereby disease was classified into stages I, II, and III when both markers were lower than prespecified thresholds, only one was elevated or both were elevated, respectively^14^. Furthermore, stage III was further divided to include stage IIIb in the event of a BNP > 700 pg/mL^14^. This allows for centers without access to NT-proBNP or TnT to accurately stage AL amyloidosis. It is evident that the aforementioned models are very successful in dividing patients into distinct survival groups when examining the 12-year survival curves for the same patient dataset^10^.

**Table 1:** Amyloidosis staging models.

Model	Criteria	Points	Score	Stage
Mayo 2004	Troponin (TnT) > 0.035 microg/L	1 point	0	Stage I
			1	Stage II
	NT-proBNP > 332 ng/L	1 point	2	Stage III
Mayo 2012	Troponin (TnT) ≥ 0.025 ng/mL	1 point	0	Stage I
			1	Stage II
	NT-proBNP ≥ 1800 pg/mL	1 point	2	Stage III
	dFLC ≥ 18 mg/dL	1 point	3	Stage IV
European 2015	Troponin (TnT) > 0.035 microg/L	1 point	0	Stage I
			1	Stage II
	NT-proBNP > 332 ng/L	1 point		
			2	Stage IIIa
	NT-proBNP > 8500 ng/L	1 additional point if score = 2		
			3	Stage IIIb
BU 2019	Troponin (TnI) ≥ 0.1 ng/mL	1 point	0	Stage I
			1	Stage II
	BNP ≥ 81 pg/mL	1 point		
			2	Stage III
	BNP ≥ 700 pg/mL	1 additional point if score = 2		
			3	Stage IIIb

## Risk-adapted management approach

When choosing the appropriate treatment approach, potential organ impairment needs to be considered. While the younger and fitter patient can tolerate more intense therapy, the frailer one has poor treatment tolerance and is, as such, at an increased risk of early mortality. The determination of frailty in amyloidosis is not accompanied by specific frailty scores, but short-term survival can be used as a surrogate to estimate frailty. Age and the number, type and extent of organ involvement and to a lesser extent, pre-existing comorbidities, are the main determinants of frailty in this patient population.

The Mayo Clinic investigators looked at the effect of age, among other factors, on survival in a cohort of 592 patients with mass-spectrometry-verified AL amyloidosis between 2008 and 2015. The cohort was followed up for 8 years and the median overall survival (OS) was 44 months. Dividing the patients into two age groups, <65 years and ≥65 years, it was evident that the younger patients had a much better survival than those aged ≥65 years^15^.

The effect of the number of organs involved on survival was investigated in the same cohort. There was a significant decrease in survival as the number of organs involved increased (*p* < 0.001)^15^. However, it is not just the number of organs involved in the disease, but also the type of organs involved that determine survival. For instance, comparing survival in patients with isolated cardiac involvement to those with cardiac plus multi-organ involvement, yields very similar survival curves between the two groups (*p* = 0.51)^15^. On the other hand, comparing survival in patients with isolated renal involvement to those with renal plus multi-organ involvement, yields significantly different survival curves (*p* < 0.001) whereby isolated renal involvement infers a survival advantage compared to the latter. As such, cardiac involvement is therefore the most important prognostic factor in AL amyloidosis whereby when present, irrespective of other organ involvement, influences survival. As previously discussed, several powerful staging models build on cardiac involvement to classify disease and aid treatment decisions.

Another important determinant of outcome and treatment response is cytogenetic profile. A recent retrospective chart review of 140 AL amyloidosis patients explored the most frequent cytogenetic abnormalities and their impact on survival^16^. Sixty-one percent of the patients harbored a cytogenetic abnormality, the most common of which was translocation t(11;14) accounting for 44%, followed by hyperdiploidy (43%)^16^. A statistically significant relationship was noted between several abnormalities and increased plasma cell (PC) burden, including gain (+) 5p/5q (*p* = 0.025), del13q (0.009), +11q (*p* < 0.001), and hyperdiploidy (*p* < 0.001)^16^. In multivariable analysis, hyperdiploidy was confirmed a significant poor prognostic factor^16^. In the setting of cardiac involvement, hyperdiploidy was also associated with worsening progression-free survival (PFS) (*p* = 0.0497) and OS (*p* = 0.006)^16^. Similarly, del13q was associated with cardiac involvement but did not appear to impact survival^16^. In addition, the overall presence of t(11;14) did not carry any prognostic value in terms of PFS and OS, although on further stratification, patients harboring isolated t(11; 14) had relatively worse PFS relative to those without any cytogenetic abnormalities^16^. Conversely, the absence of any cytogenetic abnormalities was associated with improved PFS and OS (*p* = 0.042 and 0.019, respectively)^16^. This becomes important as well when choosing therapy. For instance, patients with +1q were found to have a better response to treatment with daratumumab, a monoclonal receiving increased attention in the field^16^. As will be further discussed, patients harboring t(11;14) are more likely to have disease resistant to bortezomib-based regimens and are more likely to respond to venetoclax^17,18^.

## Treatment of ASCT-eligible patients

**Fig. 2:**
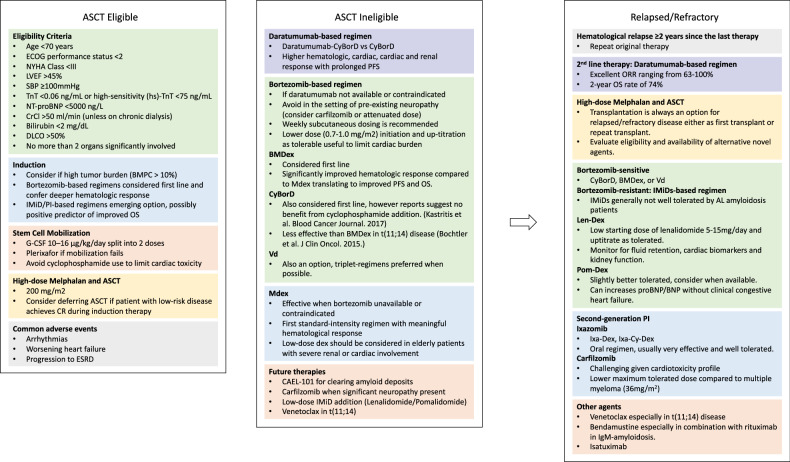
Treatment algorithm for AL amyloidosis. ASCT autologous stem cell transplantation, ECOG Eastern Cooperative Oncology Group, NYHA New York Heart Association classification of the extent of heart failure, LVEF left ventricular ejection fraction, SBP systolic blood pressure, TnT troponin T, CrCl creatinine clearance, DLCO diffusion capacity of the lungs for carbon monoxide, BMPC bone marrow plasmacytosis, IMiD immunomodulatory imide drugs, PI proteosome inhibitors, OS overall survival, G-CSF granulocyte colony-stimulating factor, CR complete remission, PFS progression-free survival, ESRD end-stage renal disease, ORR overall response rate.

The first question to answer upon considering a treatment plan for a patient is whether or not the patient is eligible for ASCT. High-dose melphalan with subsequent ASCT (HDM-ASCT) remains the standard of care in low-risk patients^19^. Approximately 20% of patients are likely eligible for this procedure. For those ineligible, a cautious approach is necessary, involving standard intensity and, for the frailer patients, low-intensity therapies (Fig. 2).

The eligibility criteria for ASCT have evolved over the years and with the emergence of novel agents^12,20–22^. Typically, potentially reversible contraindications are assessed before deeming a patient ineligible for ASCT. Otherwise, the presence of one exclusion criteria, specifically pertaining to cardiopulmonary or renal status could suffice in making a patient ASCT-ineligible. The most common eligibility criteria for ASCT are^9^:

- Age < 70 years
- Eastern Cooperative Oncology Group (ECOG) performance status < 2
- NYHA Class < III (New York Heart Association classification of the extent of heart failure)
- Left ventricular ejection fraction (LVEF) > 45%
- Systolic blood pressure (SBP) ≥ 100 mmHg
- TnT < 0.06 ng/mL or high-sensitivity (hs)-TnT < 75 ng/mL
- NT-proBNP < 5000 ng/L
- Creatinine clearance (CrCl) > 50 ml/min (unless on chronic dialysis)
- Bilirubin < 2 mg/dL
- Diffusion capacity of the lungs for carbon monoxide (DLCO) > 50%
- No more than two organs significantly involved

The choice of therapy for ASCT-eligible patients has evolved over time and has been a matter of perspective for many years. The advantages of stem cell transplantation are high response rate and response durability whereas the disadvantages are the short-term morbidity and mortality associated with it, which can be quite significant, and the fact that this therapy can only be offered to a minority of select patients. Nonetheless, complete response (CR) rates achieved with ASCT remain higher than those possible with any other treatment regimen but data from two major groups show that CR rates after ASCT are conditioning-dose dependent^23,24^. A study carried out between July 1994 and December 2008 compared HDM (200 mg/m^2^) to a reduced dose (100–140 mg/m^2^) in 421 AL amyloidosis patients. The results demonstrated a significantly higher CR rate in the high-dose group (43% versus 24%, *p* < 0.001)^24^. Similarly, between January 2000 and August 2015, a study of 457 patients using the same melphalan conditioning-dose levels, showed a significantly higher CR rate in the high-dose group (53% versus 37%, *p* = 0.003)^23^. In both studies, high-dose melphalan was associated with lower transplant-related mortality (TRM) (9% versus 14%, *p* = 0.12; 2% versus 6%, *p* = 0.01)^23,24^. The median PFS and OS were also significantly longer in the high-dose groups in both studies.

Despite the deeper responses achieved with ASCT compared to other treatment modalities, it is important to assess the durability of such a response. A recent study looking into the need for subsequent or second-line therapy after ASCT in 186 patients highlighted that in those who had survived for at least 10 years post diagnosis, 47% remained treatment-free^25^. When categorized according to initial treatment modality, ASCT treatment versus standard-intensity therapies, 58% of ASCT patients were treatment-free at 10 years compared with only 36% in the non-ASCT group (Δ 22%)^25^. As such, long‐term survivors are increasingly seen in AL amyloidosis and ASCT is associated with a more durable response compared with standard-intensity therapies.

That said, a randomized controlled trial in 100 patients performed between 2000 and 2005 by the Intergroupe Francophone du Myélome (IFM) reported evidence that argued against ASCT for AL amyloidosis^26^. It compared high-dose standard-intensity therapy (melphalan + dexamethasone) with HDM and ASCT rescue. The non-ASCT arm had an improved survival (*p* = 0.04)^26^. Although HDM followed by ASCT had been in use for more than a decade by then, some of the participating centers in the study had just started adopting HDM-ASCT as a treatment strategy, thus contributing to the study’s limitation. In addition, patient selection criteria were not well established. For example, of the 50 patients randomized to the HDM-ASCT arm, only 37 (74%) went on to receive ASCT. This translated into a high day + 100 TRM of 24% in this group^26^. Most of the remaining patients went on to have an early death^26^. Along the same lines, a systematic review of 12 studies also found no advantage of ASCT over conventional chemotherapy in improving OS in AL amyloidosis^27^. However, the evidence was weak, and the authors cautioned that further research was needed^27^.

In North America between 1995 and 2012, a randomized, multicenter study of 1536 AL amyloidosis patients at 134 centers, demonstrated that the rate of early mortality after ASCT at 30 days and 100 days progressively declined over successive time periods (1995–2000, 2001–2006, and 2007–2012)^28^. At 30 days, the TRM rates were 10%, 7%, and 4% for these time periods, respectively, and at 100 days 19%, 11%, and 4%, respectively, (*p* < 0.01)^28^. Also, centers performing more than four transplantations per year for AL amyloidosis had superior survival outcomes^28^. This data highlight the importance of center experience in achieving better outcomes in this patient population but also explain one of the potential limitations of the IFM study—relatively earlier time of investigation.

### Induction chemotherapy

Another controversial aspect of ASCT in AL amyloidosis is whether or not induction chemotherapy is required. Hwa et al. investigated the impact of induction chemotherapy on the response following transplant^29^. All patients (*n* = 415) who received ASCT within 12 months of diagnosis were included and were divided according to baseline bone marrow plasmacytosis (BMPC) level into two groups (>10%, *n* = 116 versus ≤10%, *n* = 299)^29^. Patients with a low tumor burden (BMPC ≤ 10%) did not show any difference in response rate irrespective of induction chemotherapy^29^. For patients with BMPC > 10%, the results were very different though, whereby the overall response rate (ORR) was significantly better with a near doubling of the complete response (CR) rate in patients who had received induction therapy pre-ASCT (34% versus 18%, *p* = 0.048)^29^. Afrough et al. highlighted the improved survival with pre-ASCT induction as well in 128 patients comparing conventional induction with melphalan or steroids to novel agents such as the thalidomide-analogues, the immunomodulatory imide drugs (IMiDs), and proteosome inhibitors (PI) whereby hematological, but not organ response, was significantly higher with IMiD/PI-based regimens^30^. Induction with IMiD/PI-based regimens was also found to be a significant positive predictor of OS^30^.

Multiple clinical trials have also looked into the role of bortezomib or bortezomib-based regimens in both induction and conditioning. A prospective, single-arm trial (NCT01083316) used bortezomib-dexamethasone (Vd) for induction followed by bortezomib-HDM for conditioning whereby 100% (*n* = 27) achieved hematological response (HR) with 63% CR and 37% very good partial response (VGPR) 6 months post-ASCT^31^. The cohort of patients was followed up for an extended median of 77 months whereby renal and cardiac responses occurred in 65% and 88%, respectively, at 5-years post-ASCT^31^. Median OS and PFS were not yet reached highlighting the durability of response with the incorporation of bortezomib^32^. The phase II HOVON 104 trial (NTR3220) investigated the use of four cycles of Vd induction treatment, followed by HDM-ASCT^33^. The overall HR after induction was 80% including 20% CR and 38% VGPR with improvement 6 months post-ASCT to overall HR of 86% with 46% CR and 26% VGPR. The trial confirmed the efficacy of Vd and demonstrated the amplified outcome post-ASCT. However, due to treatment-related toxicity and disease characteristics, primary endpoint could not be reached^33^.

It remains relatively unclear whether CR in response to induction alone is comparative to CR in response to induction followed by ASCT. Given the morbidity and mortality associated with transplantation, it is probably best to wait and observe rather than proceed to ASCT in a patient who has achieved CR in response to induction chemotherapy. At this point though, stem cells could be collected for future ASCT. On the other hand, in patients who achieve CR with induction chemotherapy but have a higher chance of early relapse, proceeding to ASCT may be the better option if they are eligible for it. These may be patients who have high-risk fluorescence in situ hybridization (FISH) genetics (uncommon in amyloidosis patients), such as those previously discussed, or patients with very high BMPC at diagnosis, which resembles multiple myeloma (MM) with amyloidosis.

Given the rapid progression of amyloidosis and the fact that diagnosis and subsequent treatment are a race against the clock, it is imperative that patients do not suffer further deterioration in organ function. As such, induction chemotherapy with bortezomib-based regimen should be considered for stem cell eligible patients, particularly with BMPC > 10% or in the event of a foreseeable delay of >1 month in ASCT^9^.

### ASCT-associated toxicity

The first stage in the management of toxicity in relation to ASCT is during stem cell mobilization and collection whereby low serum albumin, elevated NT-proBNP and increased septal thickening were found to be important risk factors for toxicity^34,35^ During stem cell mobilization, patients can experience various toxicities including tachyarrhythmias, thromboembolic events, weight gain (due to fluid retention), bleeding, acute kidney injury, hypertensive crises, or hypotension. To minimize risk of toxicity, it is recommended to use granulocyte-colony-stimulating factor (G-CSF) without cyclophosphamide given the increased cardiac morbidity, significantly higher number of apheresis required, increased hospitalizations and increased toxicity associated with the latter^36^. The recommended dose of G-CSF is 10–16 μg/kg/day, either as one dose or divided into two doses, 3 days before stem cell collection for an optimal total of at least 5 × 10^6^ CD34+ cells/kg^37^. The general recommendation though, is to split the dose. If patients fail stem cell mobilization, plerixafor is a well-tolerated adjuvant^36^.

The major ASCT toxicities remain cardiac arrhythmias, worsening HF, syncope, and end-stage renal disease (ESRD). Twenty-five percent of patients who have had pretransplant 24-h Holter monitoring will have evidence of nonsustained ventricular tachycardia (NSVT), a condition associated with an inferior short-term (6 months) survival^38^. On adjustment for cardiac (Mayo) stage, NSVT was actually not found to impact peritransplant mortality and pretransplant NSVT is therefore *not* an exclusion criterion^13,38^. As such, ~50% of patients will experience arrhythmias following ASCT, most of which will be low grade but with grade 3/5 arrhythmias accounting for ~10%^39^. It remains unclear whether primary AICD (Automatic Implantable Cardioverter Defibrillator) implantation will be of benefit, even among those who are at high-risk of sudden cardiac death and so, decisions should be case specific.

Worsening heart failure is another toxicity that may be encountered during transplantation and about 5% of patients will have a reduction in EF of >10%, which increases the 100-day mortality^40^. Multiple factors contribute to the heart failure risk during peritransplant in this population including electrolyte imbalance, medications, fluid overload, arrhythmia, temporary cessation of cardiac medications, sepsis, and exacerbation of organ dysfunction during transplant. It is worth noting that standard heart failure treatments such as beta blockers, calcium channel blockers and/or ACE inhibitors, cannot be used for worsening heart failure management in this patient population and so should be avoided^41^.

Another important toxicity that could complicate ASCT is ESRD. In a study examining the association between acute renal failure and mortality in AL amyloidosis during ASCT, the medical records of 408 ASCT patients between 1996 and 2010 were examined^42^. Dialysis was required by 72 (18%) patients. Eight patients started dialysis >30 days prior to ASCT (Group II), 36 started ±30 days after ASCT (Group III) and 28 initiated dialysis >1 month after ASCT (Group IV) whereby patients who were never dialyzed were assigned to Group I^42^. Median OS was not reached in Groups I and II but was 7 months in Group III and 48.5 months in Group IV (*p* < 0.001)^42^. TRM was observed in 44.4% of the patients in Group III, 6-fold higher than the next highest group with a TRM of only 3.6% (*p* < 0.001)^42^. The most common causes of TRM were cardiac and sepsis. In the multivariate analysis, only hypoalbuminemia (<2.5 g/dL, *p* < 0.001) and estimated glomerular filtration rate (eGFR) <40 mL/min/1.73 m2 (*p* < 0.001) were independently associated with starting dialysis within 30 days of ASCT^42^. The risk of dialysis increased exponentially with increasing the number of risk factors present. If none or 1 factor was present, risk of dialysis was 2% and 10%, respectively^42^. Nonetheless, the presence of both, hypoalbuminemia and eGFR <40 mL/min resulted in a 44% risk of dialysis. As such, screening with serum albumin and eGFR may reduce the risk^42^. Unfortunately, these two risk factors are also risk factors for progression to ESRD as part of the natural progression of AL amyloidosis. Thus, the decision on whether to proceed to ASCT is a difficult one. It is important to note though, that patients are likely to experience improved renal function as the disease burden decreases post induction, then making them eligible for subsequent treatment with ASCT.

In summary, HDM-ASCT is a durable and reliable treatment option that allows for deep and sustainable responses in a minority of select, eligible patients which could be preceded by bortezomib-based induction therapy when BMPC > 10% or a foreseeable delay in ASCT is expected. Despite its consistent results, treatment with ASCT is associated with a handful of detrimental adverse events including arrhythmias, worsening HF and progression to ESRD, with the introduction of novel agents, such as bortezomib, resulting in improved results and offering an alternate treatment modality.

## Treatment of ASCT-ineligible patients

The survival pattern for transplant ineligible patients has been improving over time. Trends in presentation, management and outcome among 1551 newly diagnosed AL amyloidosis patients between 2000 and 2014 were evaluated and when stratified into three eras (2000–2004, 2005–2009, and 2010–2014), survival improved significantly over time with OS rates of 25%, 46%, and 47%, respectively, (*p* < 0.001)^43^.

The main first-line options for treatment of transplant ineligible patients are (1) bortezomib-based regimens: Vd, cyclophosphamide–bortezomib–dexamethasone (CyBorD), and bortezomib-melphalan-dexamethasone (BMDex), or (2) melphalan-dexamethasone (MDex) (Fig. 2). MDex was the first standard-intensity regimen to produce a meaningful HR and was the reason for the aforementioned improved survival from 2000 to 2014. The non-ASCT first-line regimen changed over time with 65% of patients in 2010–2014 receiving bortezomib-based therapy, 79% of patients in 2005–2009 receiving MDex, and 64% of patients in 2000–2004 receiving melphalan-prednisone^43^. The rate of better than VGPR was higher in more recent periods (66% vs 58% vs 51%; *p* = 0.001), a change largely driven by improved VGPR rates in the non-ASCT population^43^. In one study by Palladini et al., 0.22 mg/kg of melphalan plus 40 mg/day of dexamethasone were given on days 1–4 in a 28-day cycle^44^. The study proved that this combination resulted in high response rates, with a 67% HR including 33% CR. Responses were durable, and the median time to response was 4.5 months^44^. This makes MDex an effective regimen, which can be used when bortezomib is contraindicated or is unavailable. However, when dealing with elderly patients or those with pre-existing severe renal or cardiac involvement, a lower dose of dexamethasone should be considered (i.e., 20 mg on days 1–4) due to the toxicities and increased fluid retention associated with dexamethasone.

Multiple studies have looked into the use of bortezomib-based regimens upfront^44–46^. Three studies, which highlight the durable HR associated with bortezomib-based regimens, are the European CyBorD collaboration study of 230 patients between 2013 and 2016^44^, a prospective observational study by the UK National Amyloidosis Center of 915 patients between 2010 and 2017^45^, and a phase III trial of MDex versus BMDex between 2011 and 2016^46^. Across these studies the CR rates on intention-to-treat (ITT) analysis ranged from 21 to 25%^44–46^. Cardiac response varied from 17 to 38% and renal response, 15 to 44%^44–46^. The median OS across these studies was >4 years and the median follow-up period ranged from 25 to 32 months^44–46^. The phase III trial (NCT01277016) comparing MDex to BMDex in newly diagnosed AL patients reported very exciting data in 109 patients recruited in Europe and Australia (56 in MDex arm, 53 in BMDex arm)^46^. Although there was no difference in the CR and partial response (PR) rates, or in cardiac and renal response rates, at the end of treatment after a median of five cycles, the overall HR rate was significantly better in the BMDex arm compared with the MDex arm (79% versus 52%, *p* = 0.002) whereas VGPR or CR was achieved in 64% of the patients on the BMDex arm versus 39% of those on the MDex arm^46^. This translated into improvements with BMDex over MDex in PFS and OS with a 2-fold decrease in mortality (HR 0.5, [0.27–0.90])^46^.

With respect to toxicity management, it is important to avoid bortezomib in the setting of pre-existing neuropathy or to opt for an attenuated dose regimen. Subcutaneous administration is recommended at weekly dosing; twice weekly dosing is not recommended and is likely to result in termination of therapy. Because bortezomib can be cardiotoxic to this patient population, initiating at a lower dose (0.7–1.0 mg/m^2^) and uptitrating as tolerable can be considered when dealing with patients with cardiac involvement. Patients should be monitored carefully for side effects and symptoms including regular monitoring for cardiac biomarkers, especially in the setting of high-risk disease. In the emergence of neuropathy or other major toxicity, the dose of bortezomib can be reduced or discontinued.

The recent ANDROMEDA phase III trial (NCT03201965) compared CyBorD to daratumumab-CyBorD in patients with newly diagnosed AL amyloidosis. Patients received weekly daratumumab in cycles 1 and 2, every 2 weeks in cycles 3–6, and every 4 weeks thereafter up to 2 years. The safety run-in of the study showed an overall HR rate of 96% without any new safety concerns beyond that already demonstrated for daratumumab in multiple myeloma (MM) and CyBorD^47^. The primary results of the recently completed trial indicated that the addition of daratumumab results in higher HR (92% versus 77%) and VGPR/CR (79% versus 49%) with better cardiac (42% versus 22%) and renal (54% versus 27%) responses and prolonged PFS. Thus, the study concluded that the addition of daratumumab to CyBorD results in both, deeper and more rapid, HR^47^. The preliminary ANDROMEDA results are thus promising and favorably comparable to ASCT.

A phase II, open-label, dose selection study (NCT04304144) evaluated the safety and tolerability of CAEL-101 in AL amyloidosis to provide a recommended dose of CAEL-101 to be given in combination with CyBorD for a planned randomized study in Mayo stage IIIa and IIIb patients^48^. CAEL-101 is an AL amyloid fibril reactive IgG1 antibody aimed at potentially clearing amyloid deposits^48^. The study enrolled 13 patients (7 heart, 3 kidney, 3 both) in a 3 + 3 dose escalation safety study whereby the maximum dose tolerated was 1000 mg/m^2^ weekly for 4 weeks and then every other week. Patients were receiving CyBorD in tandem^48^. Of the six patients receiving the maximum dose, it was well tolerated with the exception of three significant adverse events (recurrent atrial fibrillation, Clostridium difficile infection and enlarged pleural effusion)^48^. Seven patients were evaluated for organ response and 2 met criteria in the 500 mg/m^2^ cohort^48^. Given the potential for early organ response, CAEL-101 could prove groundbreaking, awaiting two recently initiated phase III trials employing the maximum dose of CAEL-101 at 1000 mg/m^2^.

## Assessing treatment response

**Fig. 3:**
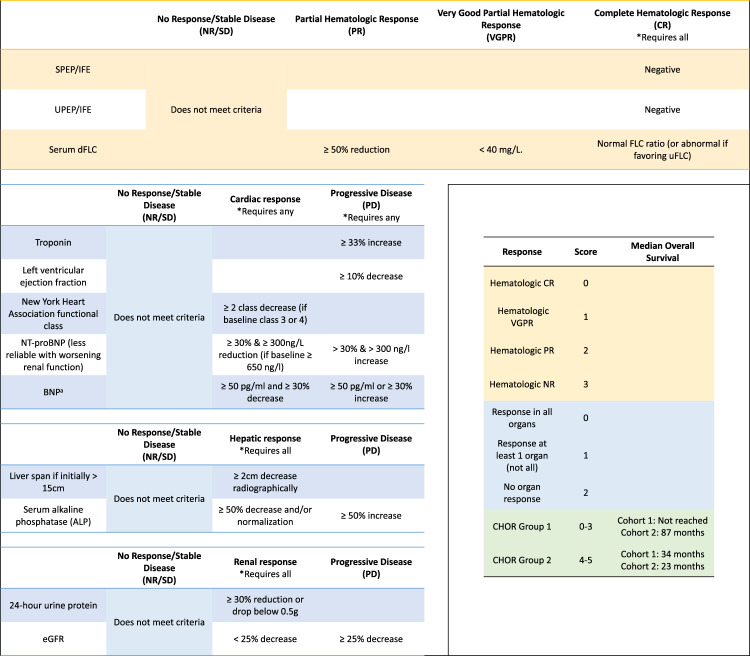
Criteria for hematologic and organ response in amyloidosis. SPEP/IFE serum protein electrophoresis and immunofixation, UPEP/IFE urine protein electrophoresis and immunofixation, dFLC delta free light chain, uFLC uninvolved free light chain, eGFR estimated glomerular filtration rate, CHOR composite hematologic/organ response model. Response criteria were derived from Palladini et al. J. Clin. Oncol 2012; Comenzo et al. Leukemia 2012; and Palladini et al. Blood 2014. a: New BNP-based cardiac criteria were derived from Lilleness et al. BJH 2020. CHOR scoring model derived from Sidana et al. Blood Cancer Journal 2020.

Palladini et al. identified the criteria for both, hematological and cardiac responses to treatment offering surrogate end points for clinical trials^49^ (Fig. 3). The analysis of a multicenter cohort of 816 patients demonstrated the strong correlation between the extent of FLC reduction and survival improvement, as early as 3 months post-treatment initiation^49^. Four levels of response/survival were identified: CR with negative serum protein electrophoresis and immunofixation (SPEP/IFE), negative urine protein electrophoresis and immunofixation (UPEP/IFE) and normal FLC ratio (FLCr); VGPR with a dFLC <40 mg/L; PR with dFLC decrease >50%; and no response (NR) with a dFLC less than that achieved in PR^49^. With the availability of highly effective antiplasma cell therapy, the criteria requirement of normal FLCr was further clarified to include abnormal FLCr inverted in favor of the nonamyloidogenic FLC^50^. As such, an abnormal FLCr does not preclude achieving CR when the uninvolved (uFLC) is greater than the involved FLC (iFLC)^50^.

In assessing treatment effectiveness, the target response for most patients should be at least a VGPR. In those with a low presenting dFLC of 20–50 mg/L, a target response should be a dFLC <10 mg/L. If a patient has a serum M-spike (true for ~50% of patients), this can help in assessing response, especially in those with a low level of circulating light chains where assessing response becomes very challenging. A retrospective analysis of 716 patients, 73% of which had a measurable M-spike before ASCT, found a measurable M-spike prior to ASCT an independent negative predictor of OS and PFS^51^. In patients with renal failure where the free light chains are cleared by the kidneys, it is sometimes hard to assess the response and a bone marrow biopsy may help to see if a deep remission has been achieved.

That said, it is empirical to determine when a shift in gears and initiation of new therapy is warranted. There are three occasions when this should be considered: (1) if there is no change in serum iFLC after the first cycle, (2) if a patient does not achieve a PR after 2–3 cycles, and (3) if treatment is not tolerated.

Increasing data are emerging about the depth of response irrespective of the HR criteria. The UK prospective study on upfront bortezomib therapy reported outcomes in the largest AL amyloidosis cohort treated with upfront bortezomib and explored the impact of post-treatment dFLC < 10 mg/L (“stringent dFLC response”) and portrayed that patients with stringent dFLC responses had significantly better OS and time-to-next treatment (TNT) compared to those with weaker responses^45^. The study looked at three levels of dFLC (<10 mg/L, 10–40 mg/L, and >40 mg/L)^45^. The median OS was not reached in patients with the first two levels and was 53 months in those with dFLC of >40 mg/L (log-rank *p* < 0.0001)^45^. The median TNT was not reached in the lowest level of dFLC, and was 38 and 13 months in the 10–40 mg/L and >40 mg/L groups, respectively (log-rank *p* < 0.0001)^45^. Cardiac responses were better in those with stringent dFLC responses (61%) compared with lesser responses (45%; *p* = 0.005)^45^. The authors concluded that a stringent dFLC response predicted prolonged TNT and impressive organ responses^45^. Similarly, in another study of patients who achieved at least a VGPR, an iFLC < 20 mg/L was associated with significantly better OS and PFS (*p* < 0.001)^52^. Using a more stringent dFLC of 5 mg/L, there appeared to be a further significant improvement in PFS (*p* < 0.001) but not in OS (*p* = 0.11)^52^. In summary, as well as using the HR criteria, a further understanding of the depth of response in a patient is possible if low levels of iFLC and dFLC are achieved.

It is important to emphasize that complete HR does not necessarily translate into organ response. As such, increasing attention is now being channeled to minimal residual disease (MRD). Sidana et al. evaluated MRD in 44 AL amyloidosis patients using MRD by next-generation flow-cytometry (NGF) at a minimum sensitivity of 10^-5^^53^. The MRD negative rate among patients in CR was 75% relative to 50% in patients achieving VGPR. Patients with MRD-negativity were more likely to have achieve cardiac response (67% vs 22%, *p* = 0.04) and improved 1-year PFS after a median follow-up of 14 months (100% vs 64%, *p* = 0.006)^53^. Similarly, Palladini et al. evaluated MRD by NGF at a minimum sensitivity of 10^−5^ in 92 AL amyloidosis patients who have achieved CR^54^. Fifty-four percent had detectable MRD. Undetectable MRD was associated with higher rates of renal and cardiac responses (90% vs 62%, *p* = 0.006 and 95% vs 75%, *p* = 0.023, respectively)^54^. Even more importantly, hematological progression was more frequent in the setting of persistent MRD (0% vs 25% at 1 year, *p* = 0.001), highlighting an important population who would benefit from further treatment^54^.

## Treatment of relapsed/refractory AL amyloidosis

Upon recognizing patients who were not able to attain a satisfactory response, it becomes vital to initiate second-line therapy as soon as possible. Currently, the recommendation for second-line therapy for relapsed/refractory AL amyloidosis (RRAL) is daratumumab or daratumumab-based therapy (Fig. 2). This is due to its association with an excellent ORR ranging from 63 to 100% whereby most responses were at least VGPR with a median time to HR of 1 week and a 2-year OS rate of 74%^51,55–58^. In a recent French study including 15 patients (median age 60 years) treated with daratumumab, ORR was 86% including 43% CR and 14% VGPR^59^. The most common side effect was infections due to induced hypoglobulinemia^59^. Thus, daratumumab has proven an excellent agent for AL amyloidosis resulting in deep remissions, high response rates and low toxicity. Nonetheless, the long-term results of daratumumab treatment and length of remission are not yet known.

Beyond daratumumab, the recommendations for third-line treatment of RRAL depend on whether the patient had a hematological relapse ≥2 years since the last therapy, in which case, the physician should consider repeating the original therapy. If the patient is not bortezomib refractory, then a bortezomib-based regimen is the preferred choice (CyBorD, BMDex, or Vd). On the other hand, if the patient is bortezomib refractory, then pomalidomide-dexamethasone (pom-dex) or lenalidomide-dexamethasone (len-dex) can be used. Importantly, ASCT can be considered in the relapsed setting, either as first ASCT or as a second transplant.

IMiDs are generally not well tolerated by AL amyloidosis patients. Whereby thalidomide is known for its high toxicity, lenalidomide comes with concerns too. The two main toxicity concerns with lenalidomide are (1) lenalidomide-related renal deterioration which, in a study of 41 patients, occurred in 66% and was only reversible in about half of them^60^, and (2) a rise in NT-proBNP/BNP. To avoid or manage this type of toxicity, we recommend a low starting dose of lenalidomide of 5–15 mg/day with the lower dose reserved for elderly patients or patients with baseline cardiac involvement or elevated creatinine. The dose can then be adjusted according to tolerability. One should observe carefully for fluid retention by weighing patients daily and measuring cardiac biomarkers as well as monitoring kidney function.

Pomalidomide is slightly better tolerated than lenalidomide and several studies have demonstrated rapid responses (median 1–3 months) and improved survival with pom-dex. A study of 33 patients with a median follow-up of 28 months highlighted the activity of the pom-dex combination even in the setting of having failed lenalidomide and bortezomib whereby the confirmed overall HR rate and PFS were 48% and 14%, respectively^61^. Median time to response was 1.9 months and the median duration of response was 19 months (95% CI, 8.3 to not attained [NA])^61^. Similarly, a phase 1/2 trial of pom-dex reported an ORR of 50% in 24 evaluable patients^62^. The median time to best ORR was three cycles, and median duration of ORR was 15 months^62^. With a median follow-up of 17.1 months, median OS had not yet been reached, and median event-free survival (EFS) was 17.8 months^62^. A phase 2 trial of pom-dex rescue treatment in 28 patients previously exposed to alkylators, PIs, and lenalidomide demonstrated a hematological ORR in 68% and a VGPR/CR in 29%, as well as improved survival with median time to response of 1 month^63^. Hematologic ORRs were also rapid, with half of responding patients doing so within 2 months^63^. Pomalidomide dose reduction was required in two out of three of those studies due to toxicity whereby patients experienced grade 3–4 myelosuppression (26–45%), fatigue (18%), pneumonia (11–21%), renal failure (3–7.5%), and arrhythmias (0–21%)^61,63^. It is important to note that AL amyloidosis patients treated with pomalidomide frequently experience increases in proBNP/BNP without clinical congestive heart failure.

PIs other than bortezomib also play a role. A few studies have looked at treatment of AL amyloidosis with the second-generation PIs, ixazomib and carfilzomib. The phase III randomized controlled trial TOURMALINE-AL1 (NCT01659658) has recently published its results. It compared the combination of ixazomib and dexamethasone (ixa-dex, *n* = 85) to physician choice (*n* = 83: len-dex, *n* = 47; melphalan-dex, *n* = 24; cyclophosphamide-dex, *n* = 10; thalidomide-dex, *n* = 2)^64^. TOURMALINE-AL1 is the first phase III trial in patients with RRAL to show a significant clinical outcome improvement. Despite the trial not meeting the first primary endpoint of hematologic ORR, treatment with ixa-dex resulted in an improved CR rate (26% versus 19%) and duration of overall HR (46.5 months versus 20.2 months) when compared to physician’s choice of treatment^64^. Although there was no difference between OS and overall PFS, treatment with ixa-dex increased hematologic/vital organ PFS and decreased time to vital organ deterioration/death, time to treatment failure, and time to subsequent therapy^64^. Considering that the duration of treatment in the ixa-dex arm was twice that of the physician’s choice arm (median 11.7 months versus 4.9 months), continuous ixa-dex was generally well tolerated^64^. When stratified by prior PI exposure, hematologic ORR was 63% versus 50% for Ixa-dex versus physician choice in PI-naïve patients and 41% versus *51%* in PI-exposed^65^. Thus, ORR was higher for Ixa-dex versus physician choice in PI-naïve patients and lower in PI-exposed (though not statistically significant)^65^. Based on these results, ixa-dex may be considered a new option for patients with RRAL, given sensitivity to bortezomib. Ixazomib was also studied in the all-oral combination, ixazomib, cyclophosphamide, and dexamethasone in a phase II trial^66^. The study included 35 patients with newly diagnosed, biopsy-proven AL amyloidosis but excluded patients with severe organ involvement (alkaline phosphatase > 750 U/L, CrCl < 30 mL/min, or NT-proBNP ≥ 7500 ng/dL)^66^. The overall HR was 57% including CR in 4%, VGPR in 26%, and PR in 17%^66^. Median PFS and OS have not been reached, four patients had disease progression and six have died^66^. Thus far, this points to modest activity in the all-oral regimen awaiting further results.

On the other hand, carfilzomib is a challenging agent due to the high incidence of cardiac involvement with AL amyloidosis. It is known that up to 10% of MM patients treated with carfilzomib experience cardiac toxicity. It is also an intravenous infusion and patients require hydration, which can aggravate patients predisposed to congestive heart failure. Results of an investigator-initiated, multicenter, phase I/II study of carfilzomib in AL amyloidosis (NCT01789242) showed that carfilzomib monotherapy was feasible and effective in 28 RRAL patients^67^. The dose escalating phase identified a maximum tolerated dose of 20/36 mg/m^2^ (which is lower than that for MM)^67^. HR rates were promising in this bortezomib-exposed population, including PI-refractory patients. The ORR and ≥VGPR were 54% and 39%, respectively, and although these are encouraging results, cardiac toxicity was experienced by 36% (including major toxicities such as ventricular tachyarrhythmia, decrease in EF, and hypoxemia)^67^.

Venetoclax is an oral, small-molecule B-cell lymphoma 2 (BCL-2) inhibitor that induces cellular apoptosis, with encouraging activity in chronic lymphoid leukemia (CLL), non-Hodgkin lymphoma (NHL), acute myeloid leukemia (AML), and a subset of MM patients whose clonal plasma cells harbor t(11;14) and/or overexpress BCL-2. Venetoclax may be useful in the management of AL amyloidosis for two reasons: t(11;14) is present in ~50% of AL amyloidosis patients^17^, and patients with this translocation are less likely to respond to a bortezomib-based regimen^17,18^. There is limited experience with venetoclax in AL amyloidosis but in 2018, Leung et al. reported on perhaps the first patient with AL amyloidosis to be successfully treated with venetoclax after his disease markers plateaued on CyBorD^18^. Sidiqi et al. have recently demonstrated efficacy and safety of venetoclax in 12 RRAL patients treated with venetoclax between January 2017 and May 2019^68^. Patients had a median of two prior lines and all but 1 had t(11;14)^68^. The dose used was 400–800 mg/d; 7 in combination with dexamethasone^68^. Eight patients were evaluable for response and the response rate was 87% comprising 3 with CR and 4 with VGPR^68^. The median time to best response was 3.4 months and median follow-up was 11.5 months, but two patients progressed at 4 and 5 months from initiation of therapy^68^. Toxicity was minimal and there were no reports of tumor lysis syndrome. Notably, in this cohort no deaths have been observed so far. Albeit a retrospective case series with a small sample size, this study suggests high efficacy and good tolerability of venetoclax monotherapy and combination therapy in patients harboring t(11;14).

Bendamustine is another agent to consider although the response rates are not very high in the AL amyloidosis population. However, for those with an IgM amyloidosis or a lymphoplasmacytic histology, in combination with rituximab, response rates are much better, and survival is improved even in heavily pretreated patients. This is highlighted by three recently published studies^69–71^. Milani et al. reported on 122 patients, 36 of whom had IgM amyloidosis^69^. With a median time to response of 3 months, the ORR to bendamustine in the whole cohort was 35%^69^. In the 12 patients with IgM-AL amyloidosis, this was achieved in 58% of subjects (21% with ≥VGPR)^69^. The median follow-up of living patients was 31 months (IQR, 17–46) and severe adverse effects were observed in 26%^69^. Lentzsch et al. conducted a phase II, multicenter trial (NCT01222260) to assess the efficacy and safety of bendamustine with dexamethasone in 31 patients with persistent or progressive AL amyloidosis after ≥1 prior therapy^70^. Dose reduction occurred in 31% of patients and 57% of patients achieved a PR or better (11% CR, 18% VGPR)^70^. The median OS was 18.2 months and the median PFS was 11.3 months^70^. Side effects were commonly myelosuppression, fatigue, and nausea/vomiting^70^. Manwani et al. reported outcomes in 27 patients identified from the UK National Amyloidosis Centre database (5 of which were RRAL) treated with bendamustine and rituximab showing an ORR of 59% (11% CR, 37% VGPR, and 11% PR)^71^. The median follow-up was 18 months and the median PFS was 34 months. Side effects comprised: GI symptoms, fatigue, cytopenias, and neutropenic fever^71^.

Finally, another anti-CD38 monoclonal antibody, isatuximab is an IgG1k monoclonal antibody with high-affinity binding to CD38 expressed on plasma cells, which has proven efficacious in MM as a single agent or in combination. The phase II trial SWOG S1702 (NCT03499808) investigates the role of isatuximab. It included 36 patients with RRAL who have received at least one previous line of therapy^72^. The median age was 70 and prior therapies included PI (89%), high-dose therapy followed by ASCT (47%), IMiDs (28%) and an anti-CD38 monoclonal antibody (6%)^72^. Nineteen patients discontinued treatment whereby the most common reasons were adverse events, disease progression, suboptimal response and COVID-19 concerns^72^. The median duration of therapy for the 17 patients currently on treatment is 11.8 months^72^. The overall HR was 77% with 3% CR, 54% VGPR, and 20% PR^72^. The majority of drug-related side effects were grade I/II infusion-related (50%), grade I anemia (25% and lymphopenia (22%)^72^. Isatuximab thus has promising efficacy with a side effect profile similar to other anti-CD38 monoclonal antibodies^72^.

## Conclusion

The presentation of AL amyloidosis could be rather deceitful due to its mimicry of various conditions. The hallmark of disease management is early diagnosis, ideally during the routine monitoring phase of high-risk patients before the onset of symptoms and irreversible organ damage. The goal of therapy in this challenging disease is at least a hematological VGPR (dFLC > 50 mg/L). Treatment should be guided by ASCT-eligibility given the robust and durable response attainable with transplant. This could be preceded by induction chemotherapy with bortezomib-based regimens especially in the setting of high disease burden or foreseeable delay in ASCT. In the setting of ASCT-ineligibility, upfront treatment with bortezomib-based regimens, now with the incorporation of daratumumab is standard. In the setting of relapse, the best response appears to be with the use of daratumumab though multiple emerging data on other novel agents including ixazomib, isatuximab, and pomalidomide are quite promising and remarkable. As we await the results of the multiple ongoing trials including the recently initiated trials involving the anti-fibril, CAEL-101, the field is still in dire need of research involving higher risk patients.
